# Minimally Invasive Versus Open Total Gastrectomy for Gastric Cancer: A Systematic Review and Meta-analysis of Short-Term Outcomes and Completeness of Resection: Surgical Techniques in Gastric Cancer

**DOI:** 10.1007/s00268-015-3223-1

**Published:** 2015-09-08

**Authors:** Jennifer Straatman, Nicole van der Wielen, Miguel A. Cuesta, Elly S. M. de Lange – de Klerk, Elise P. Jansma, Donald L. van der Peet

**Affiliations:** Department of Gastrointestinal Surgery, VU University Medical Centre, De Boelelaan 1117, ZH 7F020, 1081 HV Amsterdam, The Netherlands; Department of Epidemiology and Biostatistics, VU University Medical Centre, Amsterdam, The Netherlands; Medical Library, VU University Medical Centre, Amsterdam, The Netherlands

## Abstract

Minimally invasive surgical techniques for gastric cancer are gaining more acceptance worldwide as an alternative to open resection. In order to assess the role of minimally invasive and open techniques in total gastrectomy for cancer, a systematic review and meta-analysis was performed. Articles comparing minimally invasive versus open total gastrectomy were reviewed, collected from the Medline, Embase, and Cochrane databases. Two different authors (JS and NW) independently selected and assessed the articles. Outcomes regarding operative results, postoperative recovery, morbidity, mortality, and oncological outcomes were analyzed. Statistical analysis portrayed the weighted mean difference (WMD) with a 95 % confidence interval and odds ratio (OR). Out of 1242 papers, 12 studies were selected, including a total of 1360 patients, of which 592 underwent minimally invasive total gastrectomy (MITG). Compared to open total gastrectomy (OTG), MITG showed a longer operation time (WMD: 48.06 min, *P* < 0.00001), less operative blood loss (WMD: −160.70 mL, *P* < 0.00001), faster postoperative recovery, measured as shorter time to first flatus (WMD −1.05 days, *P* < 0.00001), shorter length of hospital stay (WMD: −2.43 days, *P* = 0.0002), less postoperative complications (OR 0.66, *P* = 0.02), similar mortality rates (OR 0.60, *P* = 0.52), and similar rates in lymph node yield (WMD −2.30, *P* = 0.06). Minimally invasive total gastrectomy showed faster postoperative recovery and less postoperative complications, whereas completeness of the resection was similar in both groups. Duration of surgery was longer in the minimally invasive group. Only comparative non-randomized studies were available, further emphasizing the need for a prospective randomized trial comparing MITG and OTG.

## Introduction

Gastric cancer is responsible for ten per cent of all cancer-related deaths worldwide, with the highest incidence rates in Eastern Asia, Eastern Europe, and South America [1]. Until this day, the only curative treatment for gastric cancer is gastrectomy with adequate lymph node dissection [2]. As (neo)-adjuvant therapy has proven to be successful, an increasing number of patients are treated this way [3, 4]. In recent years, minimally invasive techniques have gained increasing interest in the treatment of gastric cancer. The first reported minimally invasive distal gastrectomy was performed in 1994 by Kitano et al. [5], followed in 1996 by the first minimally invasive total gastrectomy for cancer by Azagra et al. [6]. Since then, several studies, and meta-analyses examined the safety and feasibility of minimally invasive gastrectomy for cancer [7–9]. The outcomes of these studies have shown promising results such as faster recovery, less pain, shorter hospital stay, improved quality of life after surgery, and above all equal outcomes of morbidity and mortality in comparison with open gastrectomy [10]. Although the results are promising, the number of studies was relatively small, their power was low, and no difference was made between types of gastrectomy, but mainly focused on distal gastrectomy or combined the different types of gastrectomies, partial, total and/or proximal [9–11]. Consequently, a heterogeneous study population was created, and as a result, outcomes are not transferable to an actual group of total gastrectomy patients [7, 12, 13].

The aim of this study is to assess evidence for a minimally invasive approach in total gastrectomy by comparing MITG to OTG with respect to operative data, conversion rate, postoperative data, morbidity and mortality, completeness of surgical resection, postoperative recovery, and long-term outcomes.

## Materials and methods

### Literature search

To identify all relevant publications, a systematic search in the bibliographic databases PubMed, EMBASE, and The Cochrane Library (via Wiley) from inception to February 5th 2015 was performed. Search terms included controlled terms from MeSH in PubMed, EMtree in EMBASE.com as well as free text terms. Free text terms were only used in The Cochrane library. Search terms expressing ‘stomach neoplasms’ were used in combination with search terms comprising ‘open surgery’ and ‘laparoscopy.’ The reference list of included articles was hand-searched for relevant publications.

### Selection criteria

Two authors (J.S. and N.W.) independently evaluated the search findings for potential eligibility for this meta-analysis using the Medline, Embase, and Cochrane databases. The inclusion criteria were as follows: (1.) Article published in English language; (2.) Only full-text articles, no abstracts, or case reports were included; and (3.) The study had to compare minimally invasive total gastrectomy (MITG) with open total gastrectomy (OTG) for cancer.

### Definitions

Operation duration was defined in minutes, and blood loss in milliliters. All studies reporting blood loss in grams were not included in the analysis of this parameter. Hospital stay and time to first flatus were reported in days. If studies reported, these parameters in hours a conversion to days would be made. Definitions of complications varied between different studies, and there was no consensus in reporting type or grade of complication such as the Clavien-Dindo grading system for the classification of surgical complications [14, 15]. Therefore, only the number of complications was reported. In-hospital mortality was defined as mortality within 30 days after surgery. Lymph node yield was measured as the mean number of resected lymph nodes with a standard deviation. Data regarding mean resection margins were also collected along with survival data. None of the studies reported neo-adjuvant treatment.

### Data extraction and quality assessment

The reviewers (J.S. and N.W.) extracted the following data from each study: first author, title of the article, year of publication, geographical region, type of study, type of gastrectomy, type of reconstruction, TNM stage, number of patients included, number of patients who underwent open gastrectomy, number of patients who underwent minimally invasive gastrectomy, operation duration, estimated blood loss, time to first flatus, time to first oral intake, length of hospital stay, percentage of postoperative complications, and percentage of mortality. Moreover, data concerning follow-up and survival were collected. All the data were reported in means and standard deviation. If the article did not report the parameters in means and standard deviations, a request for this information was sent to the concerning author and this information was received from one author [16]. Due to the difficulty of receiving raw data, the data from the published articles were used in this meta-analysis. To assess the quality of the studies, all reviewers classified the studies using the Newcastle-Ottawa Quality Assessment Scale (NOS) for retrospective cohort studies and case–control studies [17]. A maximum of nine points could be awarded, four points for selection criteria, two points for comparability, and three points for outcome. Beforehand, the criteria were discussed between the reviewers so an equal scoring method was used. In case of doubt, deliberation was conducted between the reviewers and the problem would be resolved with mutual approval. Studies achieving six or more points would be classified as high quality and were used for further analysis. Moreover, the level of evidence was assessed for each study [18].

### Statistical analysis

The meta-analysis was performed in line with the recommendations from the PRISMA Statement for Reporting Systematic Reviews and Meta-Analyses [19]. Review Manager version 5.3.3 (2014) was used for data analyses, as downloaded from the Cochrane Library. Continuous variables were assessed using the weighted mean difference. Dichotomous variables were assessed using the Odds Ratio. To account for clinical heterogeneity, the random effects model based on DerSimonian and Laird’s method was used. *P* value <0.05 was considered statistically significant.

## Results

### Study selection

**Fig. 1:**
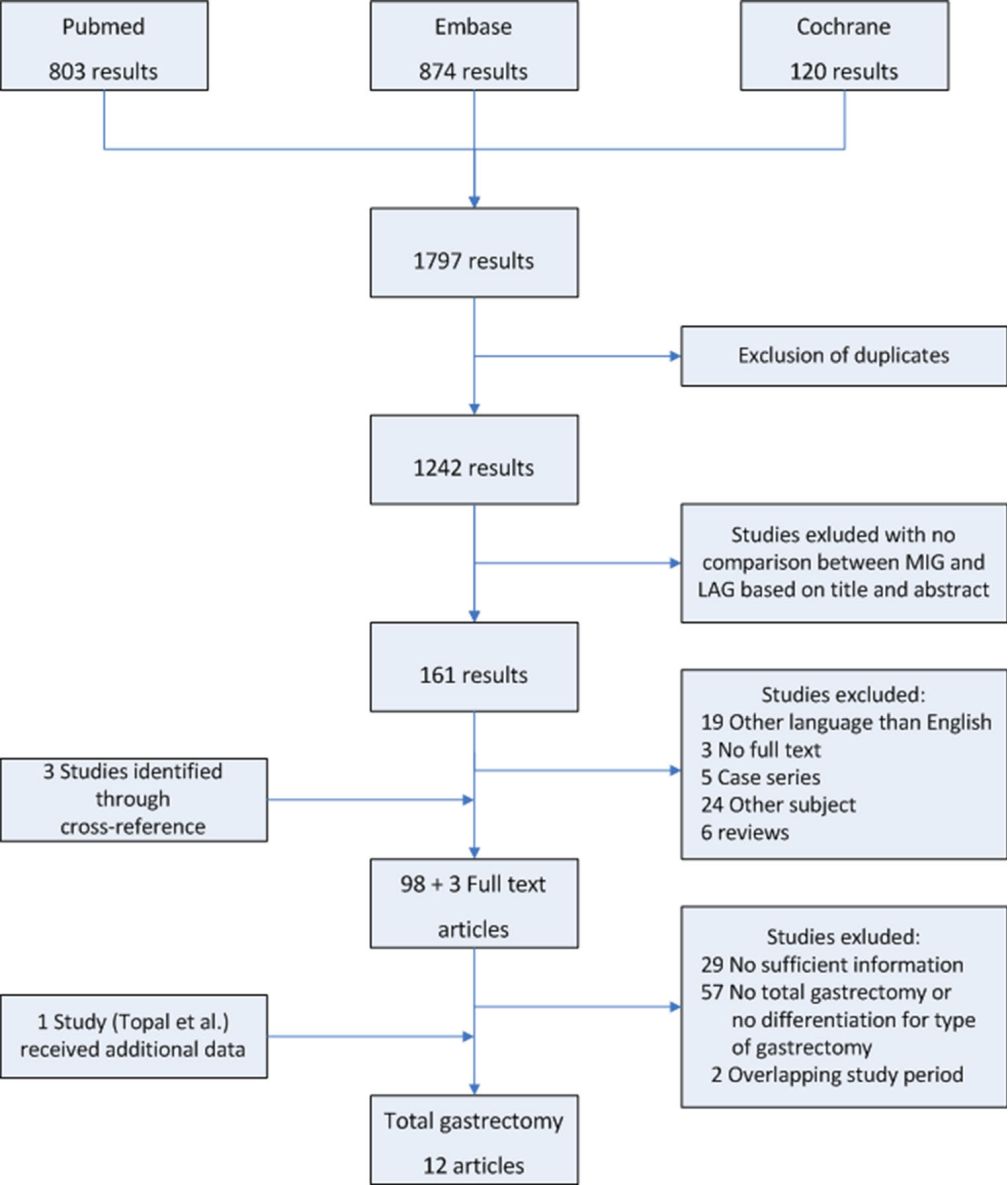
Flow-chart for selection of articles

The literature search resulted in 1797 hits; after deleting the duplicate articles, 1242 articles remained; Articles were independently screened based on title and abstract by two different authors (J.S. and N.W.) and a selection of 153 articles for full-text analysis remained. Fifty-seven articles did not meet the pre-defined criteria after reading the full-text. Via cross-referencing, an additional three articles were added, thus resulting in 99 full-text articles regarding laparoscopic versus open gastrectomy. The focus of this meta-analysis was to analyze all studies regarding total gastrectomy; therefore, we only included articles comparing minimally invasive total gastrectomy with open total gastrectomy. When the same author published more than one study from an overlapping study period, the article with the longest study period or largest cohort was included in the analysis. This eventually resulted in twelve relevant articles. A flow-chart of article selection is depicted in Fig. 1.

### Study characteristics

A total of ten retrospective cohort studies and two case–control study were identified as suitable for analysis [16, 20–30]. The total number of patients included in these studies was 1360, with 592 (43.5 %) patients who underwent MITG and 768 (56.5 %) who underwent OTG. Five studies reported that none of the minimally invasive procedures were converted to an open procedure; Lee et al. reported four patients to be converted from minimally invasive surgery to open surgery. However, these patients were excluded from the study; other studies did not report information regarding conversion rates [20, 21, 25, 27, 28, 30]. Nine out of 12 studies were conducted in Asian countries (Japan, Korea and China) and three studies were conducted in European countries (France, Italy and Belgium). An overview of the included articles is depicted in Table 1. Analysis of tumor stage according to TNM stage or tumor size showed a significant difference in two studies [25, 26]. Both studies reported a greater tumor size in the open group.

**Table 1:** Overview and characteristics of included studies

Author	Year	Study period	Design	Country	Sample size		Mean age		Sex M/F		Tumor stage (%)				Lymph node dissection
					MITG	OTG	MITG	OTG	MITG	OTG	I	II	III	IV	
Du [20]	2010	11/2005 – 05/2009	Retrospective	China	82	94	60.4 ± 18.5	57.8 ± 17.2	54/28	61/33	5.1	38.1	56.8	0	D2
Dulucq [21]	2005	04/1995 – 03/2004	Retrospective	France	8	11	75 ± 8	67 ± 14	03-May	05-Jun					D1+β
Kawamura [22]	2010	01/2003 – 12/2008	Retrospective	Japan	42	30	63.6 ± 10	64.9 ± 10.5	32/10	21-Sep					D2
Kim [23]	2008	01/2004 – 07/2006	Retrospective	Korea	27	33	57.3 ± 14.2	61.6 ± 9.2	16-Nov	23-Oct					D1+α/β/D2
Kim [24]	2011	01/2009 – 04/2010	Retrospective	Korea	63	127	55.9 ± 12.2	57.3 ± 11.1	43/20	81/46					D2
Mochiki [25]	2008	04/1998 – 12/2007	Retrospective	Japan	20	18	66 ± 2.4	63 ± 2.2	16-Apr	16-Feb					D1+β
Sakuramoto [26]	2009	07/2003 – 07/2007	Retrospective	Japan	30	44	63.7 ± 9.2	67.2 ± 9.9	Dec-18	Oct-34	54	25.7	20.3	0	D1+β/D2
Siani [27]	2012	01/2003 – 10/2009	Matched cohort	Italy	25	25	65 ± 8.5	66 ± 7.8	15-Oct	18-Jul	20	20	60	0	D1+α/β/D2
Topal [16]	2008	01/2003 – 12/2006	Retrospective	Belgium	38	22			23/15		40	23.3	26.7	10	D2
Usui [28]	2005	05/2001 – 08/2004	Retrospective	Japan	20	19	66.0 ± 10.4	66.2 ± 10.2	13-Jul	14-May	46.2	7.7	0	0	

Baseline characteristics were comparable in eleven studies. Topal et al. reported in the article that baseline characteristics were comparable; however, there was no further information regarding these characteristics [16]. Thus, this study received one point for comparability due to imprecise results. Other studies received only one point due to the fact that the study did not correct for oncological stage, which the researchers deemed to be an important factor. An overview of attributed scores is portrayed in Table 2.

**Table 2:** Newcastle-Ottowa Quality Assessment Scale score for included articles

Author	Publication year	Representativeness of the exposed cohort	Selection of the non-exposed cohort	Ascertainment of exposure	Demonstration that outcome of interest was not present at start of study	Comparability of cohorts	Assessment of outcome	Follow up long enough for outcome of interest	Adequacy of follow up	Total	Level of Evidence
						(max. 2 points)					
Bo [29]	2013	0	1	1	1	2	1	1	1	8	3
Du [20]	2010	0	1	1	1	2	0	1	1	7	3
Dulucq [21]	2005	0	1	1	1	1	1	1	0	6	3
Kawamura [22]	2010	0	1	1	1	2	1	0	0	6	3
Kim [23]	2008	0	1	1	1	2	0	1	1	7	3
Kim [24]	2011	1	1	1	1	2	1	1	1	9	3
Lee [30]	2013	0	1	1	1	2	1	1	1	8	4
Mochiki [25]	2008	0	1	1	1	1	0	1	1	6	3
Sakuramoto [26]	2009	0	1	1	1	1	0	1	1	6	3
Siani [27]	2012	1	1	0	1	2	1	1	1	8	4
Topal [16]	2008	1	1	1	1	1	1	1	1	8	3
Usui [28]	2005	0	0	1	1	2	0	1	1	6	3

### Operative results

**Fig. 2:**
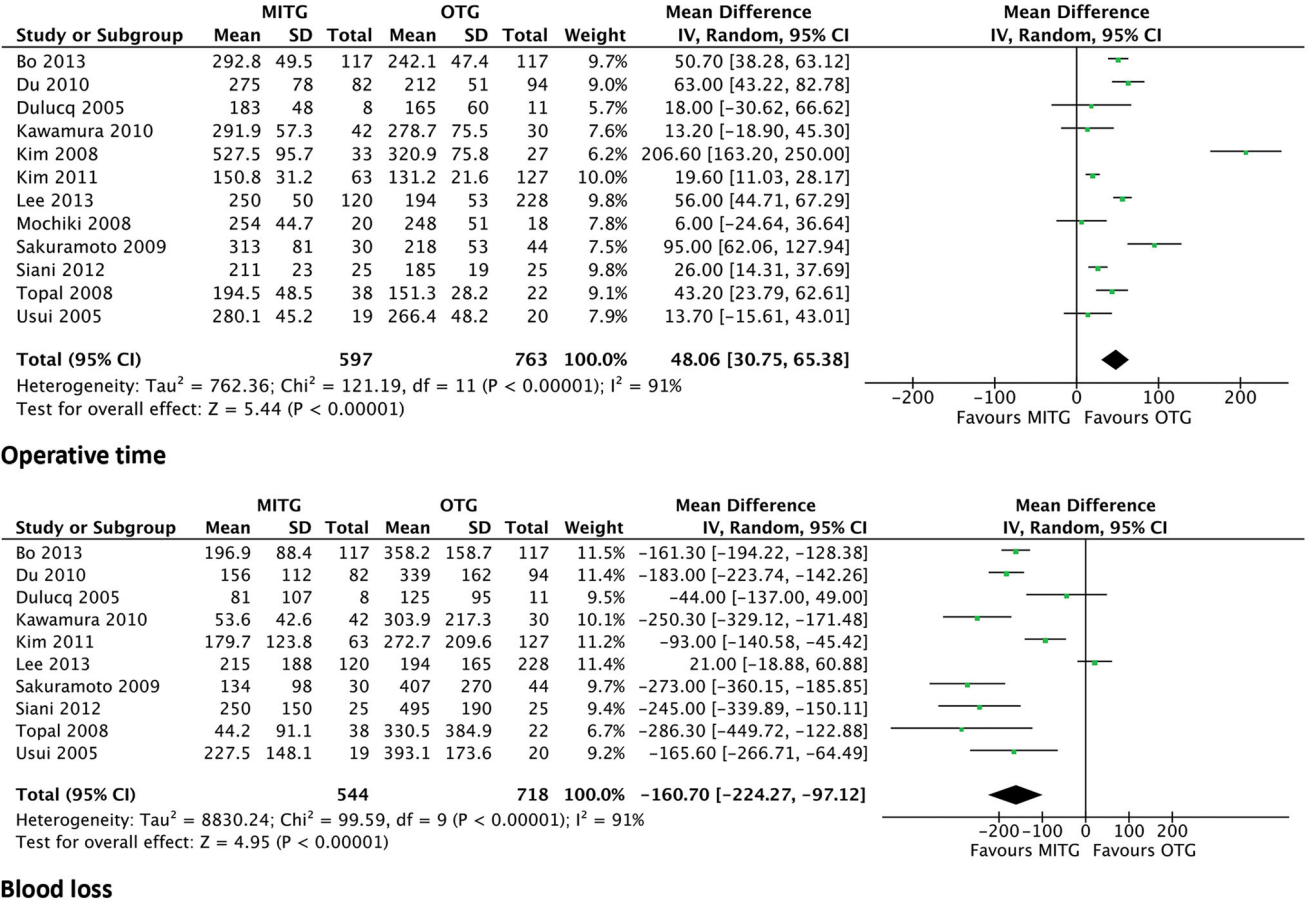
Forrest plot of comparison of operative data duration of operation (minutes) and peri-operative blood loss (ml)

All studies described the mean operative time. The procedure was found to be significantly longer for the MITG approach in all studies. The weighted mean difference was 48.06 min (95 % CI 30.75–65.38) and *P* < 0.00001 (Fig. 2).

Ten out of twelve studies described estimated blood loss. In all ten studies, blood loss was significantly less in the MITG group. The weighted mean difference was −160.70 mL (95 % CI −224.27 to −97.12 and *P* < 0.00001) (Fig. 2).

### Postoperative recovery

**Fig. 3:**
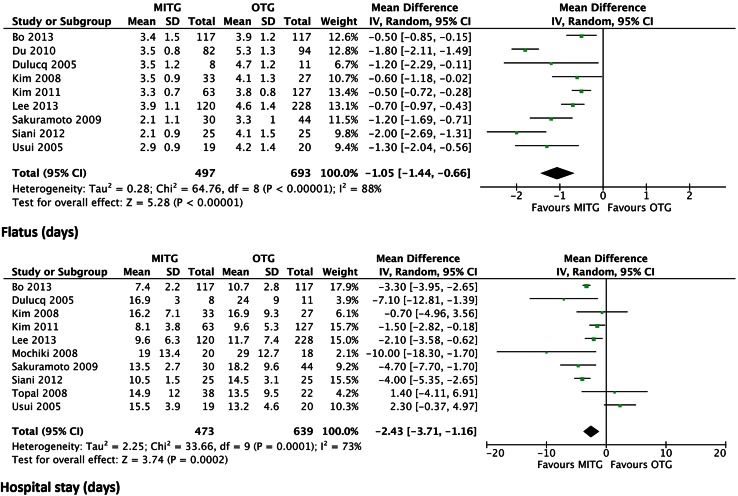
Forrest plot of comparison of postoperative recovery; time to first flatus (days); hospital stay (days)

Nine studies described the time to first flatus. The time to first flatus was significantly shorter in the MITG group. All studies showed a shorter time period to first flatus in the minimally invasive group. The weighted mean difference was −1.05 days (95 % CI −1.44 to −0.66) and *P* < 0.00001 (Fig. 3).

Ten out of twelve studies described the length of hospital stay. The duration of hospitalization was significantly shorter in the MITG group. Two studies showed a mean shorter hospital stay in the OTG group in comparison with the MITG group [16, 28]. The weighted mean difference was −2.43 days (95 % CI −3.71 to −1.16) and *P* = 0.0002 (Fig. 3).

### Morbidity and mortality

**Fig. 4:**
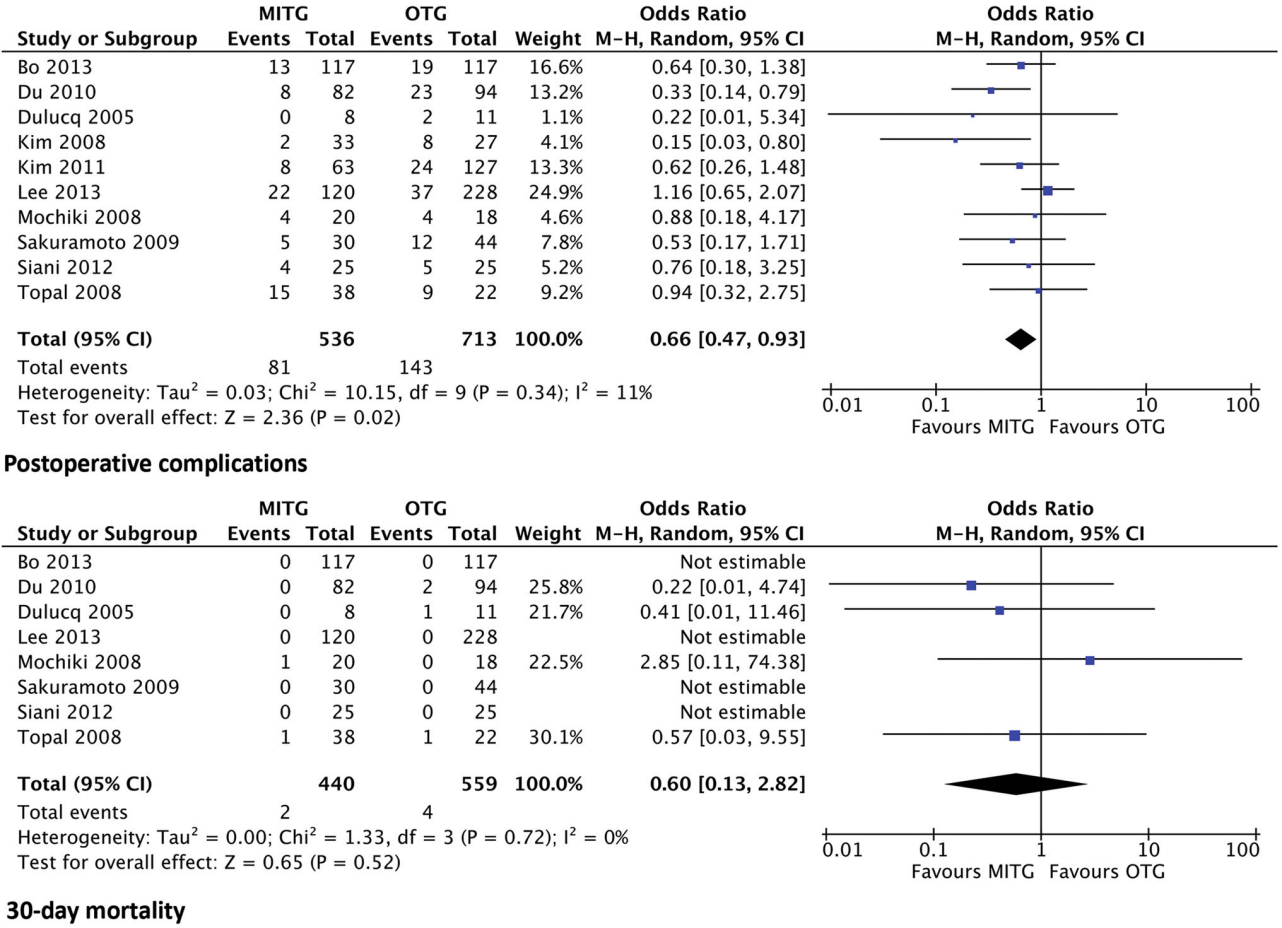
Forrest plot of comparison of morbidity with regard to postoperative complications and in-hospital mortality rates

Ten studies report results of postoperative complications. There were significantly less postoperative complications in the group who underwent MITG. The odds ratio was 0.66 (95 % CI 0.47–0.93) and *P* < 0.02 (Fig. 4). No differentiation in type of complications was listed in the included articles. Long-term follow-up data regarding complications were not available in these studies.

Eight out of twelve studies stated 30-day mortality rates, with four studies describing no mortality in both groups [26, 27, 29, 30]. Two other studies [23, 24] did not report mortality rates but did report postoperative complications. There was no significant difference in mortality rates between the MITG group and the OTG group. The odds ratio was 0.60 (95 % CI 0.13–2.82) and *P* = 0.52 (Fig. 4).

### Long-term survival

Long-term survival was reported in eight studies, ranging from 2 to 180 months follow-up. No differences in survival were reported between MITG and OTG in four studies that analyzed survival [25, 27, 29, 30]. The other articles only described survival data. Due to differences in follow-up length, differences in analysis of survival, heterogeneity between studies, and dispersion in follow-up data, pooled analysis of survival data was not possible.

### Completeness of oncological resection

**Fig. 5:**
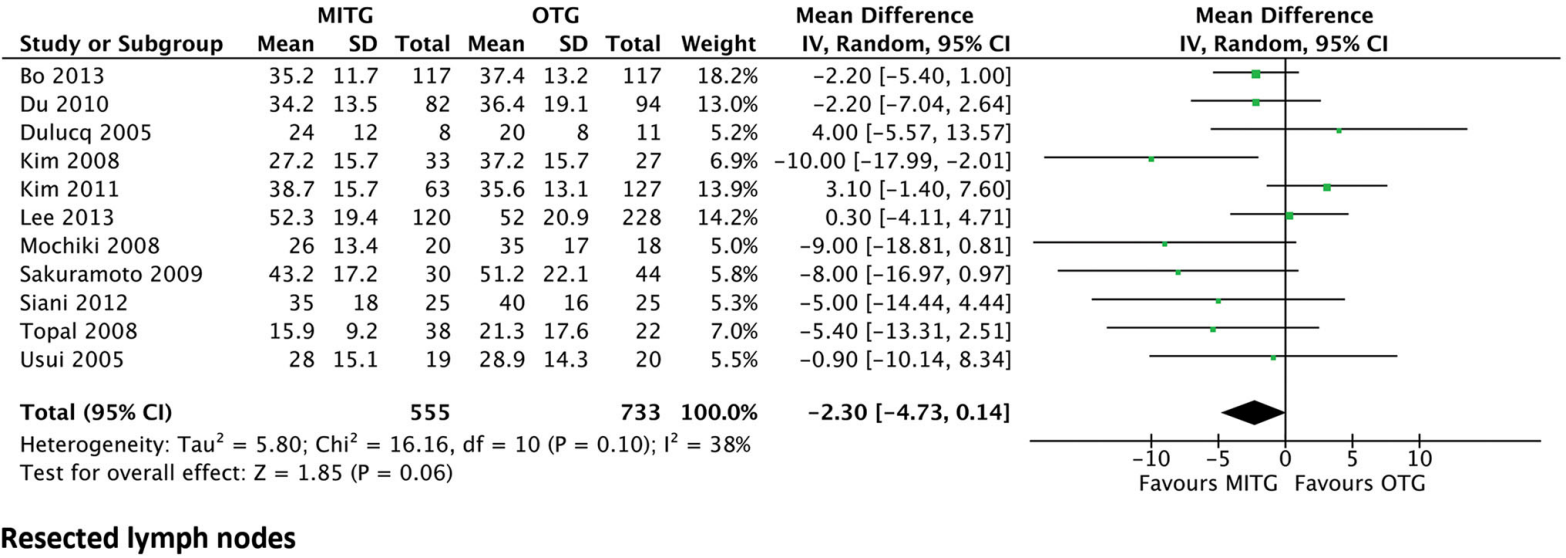
Forrest plot of comparison of number of resected lymph nodes

Eleven out of twelve studies described the total number of resected lymph nodes. There was no significant difference between the two groups. The weighted mean difference was −2.30 (95 % CI −4.73 to 0.14) and *P* = 0.06 (Fig. 5). Eight studies showed a higher mean number of resected lymph nodes in favor of the open group. None of the articles provided information on the resected stations and whether this is in accordance with a D1, D1+, or D2 lymphadenectomy.

Only three studies provided details regarding the extent of distal and proximal resection margins. No significant differences were observed in the resection margin between the groups [20, 24, 29]. Additionally, three studies reported results of resection radicality, of which two studies reported R0 resections in all patients [20, 21]. One study reported two R1 resections, one in the open group and one in the minimally invasive group; all other patients had R0 resections [16]. Thus, no significant difference was found regarding radicality of the resection in both groups.

## Discussion

The here presented meta-analysis aimed to assess the optimal surgical technique in patients undergoing total gastrectomy for cancer. The minimally invasive technique was compared to the open approach. Based on these findings, MITG was associated with less blood loss, a faster postoperative recovery, and less postoperative morbidity with similar mortality rates compared to OTG. Moreover, completeness of oncological resection concerning the number of lymph nodes resected was similar in both groups indicating that minimally invasive total gastrectomy seems to be non-inferior to open total gastrectomy in short-term outcomes. No conclusions could be made concerning long-term survival due to dispersion and inaccuracy of the disposable data.

All studies included were non-randomized and retrospective of nature. Although the observational nature might introduce bias, a meta-analysis of observational studies was deemed feasible [31]. No differences in baseline characteristics were observed in the included studies. All studies included all different stages of disease. Only two studies showed a significant difference in tumor size, where the tumor was greater in the open group [25, 26]. Most studies were conducted in Asian countries.

With exception of operative time, data concerning operative blood loss and recovery of the patient are in favor of the MITG group, indicating the reduced invasiveness of the procedure. Increased experience with this type of approach showed a clear decrease in operating time [32, 33].

This systematic review and meta-analysis assesses the optimal surgical strategy for total gastrectomy in patients with gastric cancer. Other systematic reviews and meta-analyses included both total and subtotal gastrectomy or regarded only laparoscopy-assisted techniques [9, 10].

Concerning the hospital stay, it should be noted that one article reported all patients were routinely discharged at postoperative day 14 [22]. This study was not included for analysis of length of hospital stay. Another study stated the final decision for discharge was left to the patient’s own decision [24]. No definitions of discharge criteria were provided in the other included studies. Therefore, no assurance can be made for the quality of this outcome.

Along with a faster postoperative recovery, postoperative complications were less prevalent in patients who underwent MITG. Complications were not reported using the Clavien-Dindo classification. Therefore, the grade of complications, minor or major, could not be taken into account in this meta-analysis. Also information on surgical and non-surgical complications was not provided. Moreover, no results of long-term complications or quality of life after surgery were described. Further research is necessary in order to assess the effect of minimally invasive techniques on Patient Reported Outcome Measurements such as quality of life and cost-effectiveness.

30-day mortality showed no significant differences. Data for 30-day mortality were available from eight studies. The question remains if mortality did not occur in the other study groups, if it happened past postoperative day 30, or if it was not measured at all.

The number of resected lymph nodes is considered a marker for radicality, survival, and quality of care [34–36]. A novel surgical technique should be non-inferior with regard to total lymph node resection and the distribution in stations. The here presented meta-analysis showed no significant difference in lymph node yield between MITG and OTG. There is no adequate reference to their distribution according to the Japanese classification [37]. Also information on the number of patients that received routine splenectomy was not available.

The results of the resection margin were only mentioned in three studies [20, 24, 29]. Of even greater interest is the long-term survival after both approaches with regard to survival and disease-free survival. In this meta-analysis, the analysis of long-term outcomes was not possible due to the lack of available data, heterogeneity between studies and dispersion in follow-up data. Therefore, no comparison could be made regarding this outcome.

## Conclusion

With similar results in lymph node yield, faster postoperative recovery, and less complications, the assumption may be made that minimally invasive gastrectomy is non-inferior to the open technique with regard to long-term recovery and completeness of the resection. However, resection margins and long-term survival data need to be evaluated. All included studies were non-randomized and retrospective of nature, which influences the quality of the depicted outcomes. A prospective randomized trial is indicated in order to establish the optimal surgical strategy in total gastrectomy for patients with gastric cancer and is currently underway from our department.

The primary outcome will be quality of oncological resection, as measured by the number of nodes according to the Japanese classification of gastric carcinoma, stating that minimally invasive techniques should be non-inferior. Lymph node stations are marked and analyzed separately according to the Japanese gastric cancer treatment guidelines [2]. Secondary outcomes will be postoperative recovery, hospital stay, morbidity and mortality, progression-free survival, overall survival, and quality of life [38].
